# The global cancer crisis: a review of growing burden, deepening inequality and initiatives for prevention and early detection

**DOI:** 10.3332/ecancer.2026.2071

**Published:** 2026-02-03

**Authors:** Charles M Balch, Ning Liao, Dennis S C Lam, Jeffrey N Weitzel, Rui-Hua Xu, Gerhardt Attard, Paul A Bunn, Alexander M M Eggermont, Jie He, Yuko Kitagawa, Soon Thye Lim, Eduardo Cazap, Bernard Esquivel, Xianqun Fan, Louis W C Chow, Edward S F Liu, Hector Martinez Said, John E Niederhuber, Isabel T Rubio, Ashraf Saad Zaghloul, Oscar G Arrieta, Riccardo A Audisio, Geerard L Beets, Felipe J F Coimbra, Jorge E Gallardo, Judy E Garber, Alessandro Gronchi, Volker Heinemann, Allison W Kurian, Miriam Mutebi, Masaki Mori, Funmi I Olopade, Piotr Rutkowski, Mansoor Saleh, William M Sanchez, Raymond Sawaya, John F Thompson, Gerald Tumusiime, Carlos S Vallejos, David C Whiteman, YiLong Wu, King-David T Yawe, Nayef Awad Al Zahrani, Odysseas Zoras, Banu K Arun, Carol J Fabian, Jeffrey E Gershenwald, William J Gradishar, Jin He, V Suzanne Kimberg, Ronald M K Lam, Victor H F Lee, Domenica Lorusso, Tony S K Mok, N D Perrier, Hope S Rugo, Cornelia Ulrich, Chandrakanth Are, J Vignat, I Soerjomataram

**Affiliations:** * Official Publication of the International Society of Oncology Prevention and Treatments; 1 UT MD Anderson Cancer Center, Houston, TX 77030, USA; 2 Guangdong Provincial People's Hospital, Guangzhou 510080, China; 3 Health Hope Pharma, Hong Kong SAR, China; 4 University of Kansas Comprehensive Cancer Center, Kansas City, KS 66160, USA; 5 Sun Yat-sen University Cancer Center, Guangzhou 510060, China; 6 University College Cancer Institute, London, WC1E 6DD, UK; 7 University of Colorado Comprehensive Cancer Center, Denver, CO 80220, USA; 8 University Medical Center & Princess Maxima Center, Utrecht, 3584 CS, Netherlands; 9 Cancer Hospital at the Chinese Academy of Medical Sciences, National Cancer Center of China Beijing 100021, China; 10 Keio University Cancer Center, Keio University School of Medicine, Tokyo 160-8582, Japan; 11 National Cancer Centre Singapore, Singapore 168583, Singapore; 12 Latin American School of Oncology, Buenos Aires 1426, Argentina; 13 GenXys Healthcare Systems (PGX), Vancouver British Columbia, V6B 1B8, Canada; 14 Ninth People's Hospital, Jiao Tong University, School of Medicine, Shanghai 200011, China; 15 Comprehensive Centre for Breast Diseases, UNIMED Medical Institute, Hong Kong SAR China; 16 C-MER Medical Oncology Center, Hong Kong; 17 National Cancer Institute of Mexico, Mexico City 14080, Mexico; 18 Johns Hopkins Medical Institutions, Baltimore, MD 21287, USA; 19 Breast Cancer Center, University Hospital of Navarra, Madrid 31009, Spain; 20 National Cancer Institute of Egypt, Cairo 11111, Egypt; 21 Institute of Clinical Sciences, Sahlgrenska University Hospital, University of Goteborg, Goteborg 41345, Sweden; 22 Maastricht University Medical Center, Maastricht 6202, Netherlands; 23 A.C. Camargo Cancer Center, São Paulo 1509, Brazil; 24 Cancer Institute, Catholic University of Chile, *Chilean Foundation for the Development of Oncology*, Santiago 7820436, Chile; 25 Dana-Farber Cancer Institute, Harvard Medical School, Boston, MA 002215, USA; 26 National Tumor Institute of Milano, Milan 20133, Italy; 27 Comprehensive Cancer Center, Ludwig-Maximillians University of Munich, Munich 81675, Germany; 28 Stanford Cancer Institute, Stanford University Medical Center, Palo Alto, CA 94305, USA; 29 Aga Khan University Hospital, Nairobi 30270, Kenya; 30 Tokai University School of Medicine, Tokyo 259-1193, Japan; 31 Center for Clinical Cancer Genetics and Global Health, University of Chicago School of Medicine, Chicago, IL 60637, USA; 32 Maria Sklodowska-Curie National Research Institute of Oncology, Warsaw 02-781, Poland; 33 Military Hospital of Colombia, New Granada Military University, Bogota 110111, Columbia; 34 American University of Beirut, Beirut 1107 2020, Lebanon; 35 Melanoma Institute of Australia, Sydney 2006, Australia; 36 Uganda Christian University School of Medicine, Mengo Teaching Hospital, Mukono 30055, Uganda; 37 AUNA Oncology Clinic, Lima 15036, Peru; 38 Queensland Berghofer Medical Research Institute, Royal Brisbane Hospital, Brisbane, Queensland 4006, Australia; 39 University of Abuja Teaching Hospital, Society for Oncology and Cancer Research of Nigeria, Ibadan 500107, Nigeria; 40 National Guard Health Affairs, King Abdulaziz Medical City, Riyadh 11426, *Saudi Arabia*; 41 Metropolitan Hospital, Keele University, Athens 17456, Greece; 42 Maggie Daley Center for Women's Care, Northwestern University Comprehensive Cancer Center, Chicago, Illinois, USA, IL 60611, USA; 43 University of Texas Medical Branch Cancer Center, Galveston, TX 77555, USA; 44 Department of Health, Hong Kong SAR, China; 45 University of Hong Kong, Hong Kong SAR, China; 46 Hunanitas University Hospital, Milan 20072, Italy; 47 Chinese University of Hong Kong, Hong King SAR, China; 48 City of Hope Comprehensive Medical Center, Duarte, CA 91008, USA; 49 University of Utah Huntsman Comprehensive Cancer Center, Salt Lake City, UT 84102, USA; 50 University of Nebraska Medical Center, Omaha, NE 68198, USA; 51 International Agency for Research on Cancer, World Health Organization, Lyon, France

**Keywords:** cancer, epidemiology, cancer prevention, inherited cancer, cancer vaccines, cancer screening, cancer genetics, pharmacogenomics, health care policy, health care economics, smoking, obesity, cancer viruses, breast cancer, colorectal cancer, lung cancer, gastroesophageal cancer, pancreas cancer, liver cancer, prostate cancer

## Abstract

Cancer constitutes one of the most urgent global health crises, with over 20 million new cases diagnosed annually worldwide, claiming 10 million lives. This devastating burden disproportionately affects vulnerable populations: 75% of cancer deaths occur in low- and middle-income countries despite these regions having lower reported incidence rates. Lung cancer leads the global toll, representing 12% of all cases but 19% of deaths, followed by breast cancer (12% of cases) and colorectal cancer (9.6%). A stark geographical inequity defines the cancer landscape—while wealthy regions such as North America, Northern Europe and Australia report the highest incidence rates, Africa and Latin America suffer the highest mortality rates. This disparity will intensify dramatically: cancer deaths in low-income countries are projected to surge 155% compared to just 56% in high-income nations by 2050. The financial devastation compounds this crisis, with 56% of patients worldwide facing catastrophic health expenditures extending beyond medical costs to include lost income and reduced productivity. Economic burden varies starkly by wealth: 75% of patients in low-income countries experience financial catastrophe versus 58% in middle-income countries and 26% in high-income nations. However, cancer prevention offers unprecedented opportunities to transform this crisis through interventions we already possess. From largely preventable infectious cancers like stomach and cervical malignancies, to lifestyle-driven epidemics including lung and colorectal cancers, to complex multifactorial diseases like breast cancer. Evidence-based prevention strategies can dramatically reduce suffering while generating massive healthcare savings. Emerging genetic technologies amplify this potential: universal genetic testing and pharmacogenomics now enable identification of high-risk individuals before disease develops, allowing targeted prevention while optimising treatments based on individual genetic profiles. Cancer prevention represents not merely a health opportunity but an economic imperative. The primary barrier is no longer scientific understanding but systematic implementation. Governments, policymakers, insurance companies and the public urgently need clearer evidence and education demonstrating proven successes to motivate decisive action toward creating healthier populations and reducing cancer burdens worldwide.

## Introduction 

Cancer has emerged as one of humanity's most significant health challenges, affecting every nation on earth regardless of wealth or healthcare sophistication. Today, this disease stands as the second leading cause of premature death worldwide, trailing only cardiovascular disease, but projections suggest it will claim the top position by the end of the century. The stark reality is sobering: cancer accounts for nearly one in six deaths globally. It represents one in four deaths from noncommunicable diseases, creating a crisis that touches every corner of human society.

The scale of this challenge became evident in 2022 when 20 million new cancer cases occurred worldwide, ultimately claiming 10 million lives. Behind these numbers lies a troubling pattern of inequality—71% of these cancer deaths occurred in low- and middle-income countries, revealing how the disease disproportionately devastates communities with the fewest resources to fight back. For individuals, the lifetime odds are daunting: men face a 41% chance of developing cancer and a 19% risk of dying from it, while women confront a 39% likelihood of diagnosis and 17% risk of death.

Familiar yet devastating diseases dominate the cancer landscape. Lung cancer leads both in new diagnoses, accounting for 12% of all cancer cases, and in deaths, claiming 19% of cancer victims worldwide. Female breast cancer follows closely with 12% of cancer cases, while colorectal cancer rounds out the top three with 9.6% of diagnoses. The mortality rankings tell a slightly different story, with colorectal cancer claiming 9.3% of cancer deaths, liver cancer 7.8%, breast cancer 6.8% and stomach cancer 6.8%. These patterns reveal gender-specific vulnerabilities: breast cancer dominates women's cancer experience as both the most common diagnosis and the leading cause of death, while lung cancer affects men most severely. Prostate cancer, affecting men across 118 countries as the most commonly diagnosed cancer and representing 14.2% of all diagnosed cancers, is projected to almost double from 1.47 million cases in 2022 to 2.88 million by 2050.

Five major cancer types—breast, lung, colorectal, stomach and cervical cancer—together account for almost half of the world's cancer burden , yet they present fundamentally different challenges and require tailored solutions. These five cancers reveal the multifaceted nature of the global crisis: from largely preventable infectious diseases like stomach and cervical cancer, to lifestyle-driven epidemics like lung and colorectal cancer, to the complex challenge of breast cancer that combines genetic, environmental and social determinants. Each offers distinct prevention opportunities—tobacco control for lung cancer, human papillomavirus vaccination for cervical cancer, *Helicobacter pylori* treatment for stomach cancer, lifestyle modification for colorectal cancer and early detection plus genetic risk assessment for breast cancer. Success requires coordinated action across multiple domains: tobacco control, vaccination programs, infectious disease treatment, lifestyle initiatives, early detection systems and healthcare infrastructure development. Each cancer type provides an intervention roadmap, collectively offering the possibility of dramatically reducing global cancer burden through systematic implementation of proven strategies tailored to specific disease patterns and population needs.

Geographic patterns reveal another layer of global health inequality. While the highest cancer incidence rates cluster in wealthy regions like North America, Northern Europe, Australia and New Zealand, the highest death rates occur in Africa and Latin America, illustrating how access to quality healthcare determines survival far more than disease occurrence. Adding to these concerns, younger adults under 50 are experiencing rising rates of breast, colorectal, endometrial, pancreatic and kidney cancers, suggesting emerging risk factors that could reshape cancer's future impact.^*^

In the coming decades, this crisis promises to intensify dramatically. By 2050, cancer cases worldwide are expected to reach 35.3 million—a staggering 76.6% increase from the 20 million cases reported in 2022. Even more alarming, deaths are projected to climb to 18.5 million by 2050, representing an 89.7% increase from the current 9.7 million annual deaths. This surge will affect virtually every nation, with 181 of 185 countries and territories expected to see increases, and approximately half facing doubled cancer rates within the next 25 years.

The burden will fall most heavily on those least equipped to handle it. Low-income countries face a devastating 2.5-fold increase in cancer cases by 2050—a 151% rise compared to just 39% in high-income nations. The mortality projections are even more stark: cancer deaths in low-income countries are expected to surge by 155% compared to 56% in high-income countries. This disparity underscores how healthcare infrastructure, early detection capabilities and treatment accessibility fundamentally determine whether cancer becomes a manageable disease or a death sentence.

Beyond the human toll, cancer creates financial devastation that ripples through families and societies. An estimated 56% of cancer patients worldwide face catastrophic health expenditures that extend far beyond medical bills to include lost income, reduced productivity and ongoing care costs. This financial burden varies dramatically by geography: 75% of patients in low-income countries experience catastrophic expenses, compared to 58% in middle-income countries and just 26% in high-income nations. For many families, these overwhelming costs force impossible choices between lifesaving treatment and basic survival needs.

The social consequences extend beyond individual families to reshape entire communities. Cancer's toll on mothers has created an estimated one million maternal orphans—children who have lost their mothers to this disease. This statistic represents not just individual tragedy but a broader social disruption as families fragment and communities lose vital members who would have contributed to society during their prime adulthood.

## Mapping the global cancer landscape: geographic disparities and prevention opportunities

With the world’s largest population and the country's rapid population aging, China accounts for 24% of all cancer cases worldwide, ranking 64th out of 185 countries in terms of age-standardized incidence rates. In 2022, there were 4.82 million new cancer cases and 2.57 million deaths. The top five cancer sites included the lung, colon-rectum, thyroid, liver, and stomach, whereas the top five cancer death sites were the lung, liver, stomach, colon-rectum, and esophagus. China is facing a shifting cancer burden. The age-standardized incidence rate increased by about 1.4% per year from 2000 to 2018. However, the age-standardized mortality rate decreased by 1.3% per year, mainly due to decreases in upper digestive tract cancers.

Nearly half of all cancer deaths in Chinese adults were preventable. Lifestyle risk factors such as smoking, obesity, and physical inactivity remain highly prevalent. By stage at diagnosis, 52.8% of five common cancer cases were diagnosed at a late-stage at diagnosis in China. In 2019–2021, the age-standardized 5-year relative survival for all cancers combined was 43.7%. Notable survival improvements were seen for cancers of the lung, prostate, bone, uterus and breast, with narrowing survival gaps between urban and rural areas. The improvement in cancer survival is probably due to earlier diagnosis and advances in the quality of cancer care. However, survival gaps still exist between Chinese patients and those from developed countries such as the USA and the UK, especially for cancers such as female breast, colon-rectum, and prostate. These statistics highlight the importance of strengthening comprehensive control and prevention in the battle against cancer in China.

### Asia: the epicentre of global cancer burden

Asia bears the largest share of the world's cancer crisis, housing 59% of the global population while accounting for 49% of all cancer cases and a devastating 56% of cancer-related deaths worldwide. This huge volume of cancer patients illustrates the unique challenges facing Asian healthcare systems, where population density, environmental factors and varying levels of healthcare infrastructure create a perfect storm for cancer proliferation.

India ranks third globally in cancer incidence (behind China and the United States), with over 1.4 million new cancer cases diagnosed annually. The country holds the second position worldwide for cancer-related deaths, behind China, with approximately 917,000 cancer-related mortalities occurring each year. Breast cancer emerges as the most prevalent malignancy, representing 13.6% of all new diagnoses. This is followed by oral cancer (10.2%), cervical cancer (9.0%), lung cancers (5.8%) and esophageal cancer (5.0%). These same five cancer types also constitute the leading causes of cancer-related deaths, with breast cancer alone accounting for more than 10% of all cancer mortalities.

Male cancer patterns in India have a distinctly different profile. Oral cancer predominates as the most frequently diagnosed malignancy in men, comprising 15.6% of all new cases, followed by lung (8.5%), esophageal (6.6%), colorectal (6.3%) and stomach cancers (6.2%). This hierarchy is mirrored in mortality statistics, where oral cancer accounts for 12.7% of all male cancer deaths, followed by lung (11.5%), esophageal (9.2%) and stomach cancers (8.2%). As India's demographic profile shifts from a predominantly reproductive-age population toward middle-aged and geriatric cohorts, the country is projected to experience an unprecedented cancer burden in the coming decades, potentially reaching historic levels.

Beyond China and India, stomach cancer reveals Asia's broader regional patterns, with Eastern Asia reporting 520,000 cases—the highest regional burden globally. China, India and Japan together account for 57% of the 1 million stomach cancer cases globally and 55% of 660,000 deaths, demonstrating how certain cancers cluster geographically, demanding region-specific prevention and treatment strategies. Over one-third of East Asian populations (Japanese, Chinese, Korean) have an inherited an ALDH2 deficiency that transforms alcohol from a social beverage into a cancer catalyst, dramatically increasing esophageal cancer risk with every drink. This genetic reality means that standard alcohol consumption guidelines developed for Western populations become dangerously inadequate for millions of East Asians.

In the prosperous cities of Singapore and Hong Kong SAR, China, cancer has become the leading cause of death, accounting for 25% of all fatalities and surpassing deaths from both heart disease and stroke. Despite world-class healthcare infrastructure, both regions face significant prevention challenges. Singapore's national screening data reveals a concerning gap: only 45% of eligible women had Pap tests, 35% had mammograms and just 42% of eligible adults had been screened for colorectal cancer. In response, Singapore has launched a comprehensive national prevention strategy that empowers individuals to take proactive health steps while improving access to screening programs and exploring population-based genetic testing.

Hong Kong has implemented a similar multipronged approach, emphasising evidence-based screening and healthy lifestyle promotion as primary prevention strategies. The territory has achieved notable success in tobacco control, reducing smoking prevalence to just 9.4% through concerted societal efforts. Hong Kong's Health Behaviour Survey shows modest improvements in screening participation: 46% for colorectal cancer, 50% for cervical cancer and 37% for breast cancer screening. Encouragingly, despite rising cancer incidence due to population aging, both cities have achieved declining cancer mortality rates over the past decade, demonstrating that coordinated prevention efforts can yield measurable public health benefits.

### North America: economic burden and prevention initiatives

Cancer stands as America's second-leading killer, claiming one in every six lives and revealing a troubling national paradox. While the United States represents just 4% of the world's population, it shoulders a disproportionate 11.9% of global cancer cases—2.38 million new diagnoses and 600,000 deaths in 2022 alone. This outsized burden reflects more than demographics; it exposes the unique risk landscape of American society, where lifestyle, environment and healthcare access converge to create distinct cancer patterns.

Four cancers dominate America's oncological landscape, each telling a story of the disease's complexity. Breast cancer leads with 274,000 new cases annually, followed closely by prostate cancer (230,000 cases), lung cancer (226,000 cases) and colorectal cancer (160,000 cases). However, these incidence numbers mask a more sinister reality.

The true measure of cancer's devastation lies not in diagnosis rates but in mortality, where lung cancer emerges as America's deadliest adversary. Despite ranking third in new cases, lung cancer claims 128,000 lives annually—the equivalent of breast, prostate and colorectal cancers combined. This stark disparity between incidence and mortality exposes lung cancer's particularly lethal nature, where late detection and expensive treatment options transform what might be manageable diagnoses into death sentences. The paradox is that this leading cancer killer is preventable or even curable if detected before symptoms arise, by computed tomography scanning.

America's cancer burden extends far beyond hospital walls, creating an economic crisis that rivals any national emergency. The numbers tell a stark story: $157.7 billion in direct medical costs annually, plus another $95 billion in lost productivity. Looking ahead, economists project a devastating cumulative impact of $25.2 trillion between 2020 and 2050—effectively transforming cancer from a health challenge into an existential threat to America's economic future. Just two cancers illustrate this massive burden: breast cancer market was valued at $34.6 billion in 2024 (14% of all cancer costs)—and is projected to reach a staggering $89 billion by 2034—while colorectal cancer consumed $24.3 billion (12.6%). Targeted therapies, immunotherapy and new diagnostic technologies are fueling the growth.

However, America's cancer story also highlights remarkable prevention successes that offer hope and a roadmap forward. The clearest victory comes from tobacco control. Since smoking causes 85%–90% of lung cancers, aggressive cessation policies and targeted screening have delivered dramatic results: lung cancer death rates have plummeted 30% for men and 20% for women since 2000. Breast cancer presents an even more compelling success story—mammography, awareness campaigns and improved treatments have slashed death rates by 40% over four decades, creating a prevention model that other nations now emulate. Similar gains appear across multiple cancers: cervical cancer deaths have declined every year since 2003 thanks to screening and human papillomavirus (HPV) testing, while both prostate and colorectal cancer deaths have dropped 30% since 2000.

However, enormous untapped potential remains. Despite proven screening benefits, only 63% of eligible Americans stay current with colorectal cancer screening. Achieving just 80% compliance could prevent 22% of new cases and eliminate 33% of deaths. The stakes are literally life and death: early-stage colorectal cancer patients enjoy an 88% 5-year survival rate, compared to merely 16% for those diagnosed late. More troubling, colorectal cancer rates are actually rising among adults under 40 years of age, outside of the standard guidelines for initiating screening.

### Latin America: an escalating cancer crisis

Latin America and the Caribbean confront a rapidly intensifying cancer epidemic that threatens to overwhelm healthcare systems across the region. Cancer has already emerged as the most frequent cause of premature death in most countries of the region and ranks as the second leading cause of death overall, regardless of national economic status. With cancer incidence climbing annually, projections indicate the region will face 2.6 million new cancer cases by 2045—a 69% surge from current levels. The window for prevention is rapidly closing.

Every healthcare system in the region currently grapples with this cancer tsunami of over 1.5 million new cancer cases and 747,000 deaths each year—numbers that represent not just statistics, but families shattered, and economies drained. Prostate cancer leads the charge, representing 15% of all cases, followed by breast cancer (14%), colorectal cancer (9%), lung cancer (7%) and stomach cancers (5%). However, these incidence patterns tell only part of the story—lung cancer, despite ranking fourth in frequency, is the region's deadliest malignancy, responsible for 12% of all cancer deaths.

The 2022 GLOBOCAN estimates reveal stark disparities across the region. Cumulative cancer incidence risk ranges dramatically from a modest 11.6% in Belize to a staggering 27.8% in Uruguay, while mortality rates span from 6.1% in Belize to 13.7% in Uruguay. These variations expose profound inequalities in healthcare access, treatment quality and prevention infrastructure across Latin American nations.

The region's cancer outcomes paint a complex picture of progress and persistent challenges. For breast and prostate cancers, 5-year survival rates exceed 80% in most countries, reflecting advances in early detection and treatment. However, this success story contrasts sharply with other malignancies. Colon and cervical cancers, along with lymphoid malignancies, achieve survival rates of only 50%–60%, while lung and stomach cancers, along with myeloid neoplasms, fall below the 50% threshold. Remarkably, except for uterine, stomach and prostate cancers, cancer mortality rates remain relatively low throughout Latin America. This pattern suggests significant opportunities for improvement through targeted interventions, including tobacco control, organised cervical cancer screening programs and enhanced treatment protocols, particularly for prostate cancer.

Mexico's cancer landscape reflects unique regional health care challenges for early detection and treatment. While breast cancer dominates new diagnoses at 15%, followed by prostate (12.8%), colorectal (7.8%), thyroid (5.5%) and cervical (5%) cancers, the mortality picture tells a more troubling story. Colorectal cancer becomes Mexico's leading killer at 8.6% of deaths, closely trailed by breast cancer (8.5%), lung cancer (8.1%), liver cancer (8%), prostate cancer (7.6%) and stomach cancer (7.5%). Most striking is cervical cancer's persistence among the top five diagnoses—a disease that should be virtually eliminated in any nation with effective HPV prevention and screening programs.

Argentina faces a distinct cancer profile dominated by breast cancer (16%), followed by colorectal (12%), lung (10%), prostate (9.6%) and pancreatic (4.2%) cancers. But the mortality statistics reveal tobacco's deadly impact: lung cancer claims 15% of all cancer deaths—among the highest proportions in the region —followed by colorectal cancer (12.5%), breast cancer (9.2%), pancreatic cancer (7.3%) and prostate cancer (6.2%). This mortality pattern reflects Argentina's struggle to implement effective tobacco control measures and address air quality challenges that continue claiming lives long after exposure ends.

Brazil's cancer burden mirrors broader regional patterns. Prostate cancer leads the incidence at 16%, followed by breast (15%), colorectal (9.6%), lung (7%) and thyroid (5%) cancers. However, the mortality profile shifts dramatically, with lung cancer claiming 13.7% of deaths, followed by colorectal (10.4%), breast (8%), prostate (7.2%) and stomach (6.5%) cancers. Brazil exemplifies the diagnostic challenges plaguing Latin American healthcare systems, particularly evident in stomach cancer cases, where the majority of patients receive diagnoses at advanced disease stages III or IV, resulting in devastating survival outcomes. The stomach cancer crisis in Brazil also highlights nutritional factors contributing to cancer risk. Added sugar and increased salt intake have been directly linked to gastric cancer development, pointing to the need for comprehensive dietary interventions alongside medical treatment improvements.

Latin America exemplifies some of the world's most striking cancer patterns—geographic clusters that reveal how ethnicity, lifestyle and environment converge to create cancer hotspots, while simultaneously demonstrating the extraordinary power of targeted public health interventions to save lives. The Andean corridor—encompassing Chile, Bolivia, Peru and Argentina's mountainous regions—once held the grim distinction of hosting the world's highest gallbladder cancer rates. This deadly clustering was not random but reflected a perfect storm of risk factors: gallbladder stones plagued indigenous populations at extraordinary rates, compounded by poverty and rising obesity. For generations, gallbladder cancer reigned as the leading cause of cancer deaths among women in these nations. Systematic implementation of prophylactic cholecystectomy—preventive gallbladder removal in a high-risk population—has helped mortality rates plummet by an astounding 65% in just two decades. What was once a common killer is now a rare event, proving that when public health policy targets the right intervention at the right population, even the most entrenched cancer patterns can be altered.

The same Andean-Pacific region has long suffered from among the world's highest gastric cancer rates, creating another deadly ‘geographic hot spot’. This cancer's persistence reflects a complex web of risk factors: endemic *H. pylori* bacterial stomach infection, traditional diets heavy in salt and smoked foods, limited access to fresh vegetables and fruits and poverty that made healthy choices impossible for millions. For decades, gastric cancer dominated as the leading cause of cancer death among men throughout the region. However, here too, targeted intervention has yielded extraordinary results. Through systematic *H. pylori* eradication antibiotic programs and improved nutrition access, gastric cancer mortality has been slashed by more than 50% over 20 years.

### Europe: a disproportionate cancer burden of west and east

Europe confronts a cancer crisis that far exceeds its global demographic footprint. Despite housing less than 10% of the world's population, the continent bears 22% of global cancer cases and 20% of cancer-related deaths—a disproportionate burden that reflects complex interactions between aging populations, lifestyle factors and healthcare disparities across the region.

In 2022, Europe recorded 4.47 million new cancer cases . With an age-standardised incidence rate of 280 per 100,000 people, Europeans face a cumulative cancer risk of 27.9% by age 75—meaning more than 1 in 4 Europeans will develop cancer before their 75th birthday. The human toll is staggering: 2 million cancer deaths annually, with cancer the leading cause of death among Europeans under 65 years old. The economic burden mirrors the human cost, with cancer consuming nearly €97 billion across the European Union in 2018. Projections paint an even more challenging future, with new cases expected to reach 5.33 million annually by 2040, translating to over 100 million new cancer patients over the next two decades.

Cancer strikes European men more frequently and fatally than women. Males account for 53% of new cases (2.36 million) and 55% of deaths (1.1 million), facing a cumulative risk of 31.9% by age 75 compared to 24.7% for women. The mortality disparity is even starker: 1 in 7 men will die from cancer before age 75, compared to 1 in 11 women. Age dramatically amplifies cancer risk across Europe. While cancer predominantly affects older adults—with 64% of diagnoses and 74% of deaths occurring in those 65 and older—the disease increasingly impacts younger populations, with 30% of diagnoses and 23% of deaths affecting people aged 45–64.

Europe's cancer landscape reveals a striking paradox that defines the continent's health challenges. Northern European countries, led by Denmark, report the highest cancer incidence rates—a phenomenon that initially appears alarming but actually reflects sophisticated healthcare systems. These elevated rates stem from comprehensive screening programs, advanced detection methods and heightened public awareness that catch cancers early when they are most treatable.

Eastern Europe presents the opposite reality: lower reported incidence rates mask a more sinister truth. Limited healthcare investments, restricted access to screening programs and challenging lifestyle factors create an environment where cancers often go undetected until advanced stages. This geographic disparity illuminates a fundamental healthcare equity crisis across the continent. Cancer mortality patterns starkly reverse the incidence trends, exposing the true impact of healthcare disparities. Eastern Europe records the highest mortality rates, particularly among men, with Hungary bearing the devastating distinction of having Europe's highest cancer death rate.

Certain European nations demonstrate how comprehensive cancer prevention strategies can dramatically alter outcomes, providing blueprints for continental improvement. For example, Finland’s organised colorectal cancer screening program, utilising fecal immunochemical tests, has achieved measurable reductions in mortality rates. This systematic approach proves that even relatively simple screening technologies can save lives when implemented comprehensively across populations. Sweden's national breast cancer screening program exemplifies how sustained investment in early detection translates directly into improved survival outcomes. The program's success in identifying cancers before they become symptomatic has contributed significantly to declining breast cancer mortality rates. Both the Netherlands and the United Kingdom have established gold-standard colorectal cancer screening programs that have led to declining mortality rates, demonstrating how systematic screening can bend the cancer mortality curve when properly implemented and funded.

However, despite overwhelming evidence supporting cancer screening effectiveness, Europe's implementation remains fragmented. The disparities are particularly stark when examining specific programs. For example, while most Western and Northern European countries maintain organised programs, participation rates are dramatically lower in Eastern European regions due to financial barriers, lack of awareness and insufficient healthcare resources. Countries like the Netherlands, UK and France have established comprehensive nationwide programs, whereas many Eastern European nations still lack systematic implementation. The cumulative impact of these screening gaps creates a two-tiered system where geographic location determines cancer survival prospects.

### Africa: systemic challenges and preventable solutions

Africa confronts perhaps the most tragic manifestation of the global cancer crisis—a continent where the disease burden far exceeds the healthcare capacity to address it, transforming manageable conditions into death sentences. The region faces a devastating epidemiologic transition, shifting from infectious diseases toward noncommunicable diseases while simultaneously experiencing a projected doubling of cancer incidence. This transition occurs against a backdrop of inadequate healthcare infrastructure, creating a perfect storm where approximately one-third of cancers remain attributable to infectious agents that could be prevented or treated with proper resources.

Africa's cancer crisis manifests most starkly through a fundamental disparity: the continent bears a disproportionately high cancer mortality burden relative to its reported incidence rates. While cancer cases are widely acknowledged to be underreported across Africa—due partly to incomplete surveillance systems and limited diagnostic capacity—the available data reveals that African cancer patients face dramatically higher death rates compared to patients in other regions. This striking mortality-to-incidence gap exposes critical systemic failures in early detection, treatment access and healthcare infrastructure that systematically transform potentially curable diseases into fatal diagnoses. The pattern suggests that not only are many cancers going undiagnosed, but those that are identified often receive inadequate or delayed care, creating a devastating cycle where Africa simultaneously underdiagnoses cancer while experiencing some of the world's worst cancer outcomes.

Cervical cancer represents the most devastating example of Africa's preventable cancer crisis. Sub-Saharan Africa bears the highest cervical cancer rates globally, with the disease ranking as the most frequent cancer type in Eastern Africa and the leading cause of cancer deaths in Eastern and Middle Africa. In 29 of the 48 sub-Saharan African countries, cervical cancer stands as the most common cause of cancer death among women—a statistic that should shock the global health community.

Africa's cancer profile remains uniquely shaped by infectious agents, with approximately one-third of cancers attributable to preventable infections. Cancers of the cervix, stomach and nasopharynx rank among the most common, reflecting the persistent impact of HPV, *H. pylori* and Epstein-Barr virus, respectively. The preventable nature of this crisis amplifies its tragedy. Close to 100% of cervical cancer cases are attributable to HPV, making every death theoretically preventable through vaccination and screening programs. However, fewer than 10% of women aged 30–49 years have ever received cancer screening in sub-Saharan Africa, compared to over 80% in most Western countries. This screening gap represents one of the most profound healthcare equity failures of our time.

Breast cancer in Africa illustrates how inadequate surveillance systems can mask true disease burdens. Sub-Saharan Africa experienced a staggering 247% increase in breast cancer incidence from 1990 to 2019, with deaths increasing by 184% over the same period. Nigeria recorded the highest regional incidence, while central sub-Saharan Africa shows the fastest-growing breast cancer rates on the continent.

Esophageal cancer presents a particularly striking challenge in Kenya, ranking as the second most common cancer diagnosed in men and the third most common in women. Kenya reports one of the world's highest esophageal cancer incidence rates, likely linked to the consumption of extremely hot foods and beverages combined with alcohol use. This pattern suggests that relatively simple behavioural interventions—avoiding very hot food and beverage consumption and reducing alcohol intake—could significantly reduce esophageal squamous cell carcinoma (SCC) rates across East Africa.

Cancer registry data from four major African population centres—Nairobi (Kenya), Eastern Cape Province (South Africa), Kyadondo County (Uganda) and Harare (Zimbabwe)—reveal distinct patterns that reflect both infectious disease legacies and emerging lifestyle-related cancers. Among older men, prostate and esophageal cancers dominate the disease landscape, while older women primarily face breast, cervical and esophageal cancers. These patterns reflect Africa's unique position in the global cancer transition, where traditional infectious agent-related cancers persist alongside emerging lifestyle-associated and non-viral related malignancies.

The Middle East and North Africa region confronts an escalating cancer crisis, with breast and colorectal cancers showing some of the steepest projected increases worldwide. This mounting burden carries substantial economic implications that continue to expand. Urinary bladder cancer (UBC) presents a particularly distinctive epidemiological profile in the region. Several Arab countries exhibit disproportionately high UBC incidence rates driven by region-specific risk factors, notably schistosomiasis and elevated smoking prevalence. In Egyptian males, UBC ranks among the most prevalent malignancies—historically linked to schistosomiasis, a primary driver of SCC. However, recent epidemiological data reveal a significant shift: transitional cell carcinoma incidence is rising due to increased smoking rates, while SCC cases are declining as schistosomiasis prevention efforts take effect.

### Australia: global melanoma capital and prevention pioneers

Australia and New Zealand occupy a unique position in the global cancer landscape, simultaneously facing the world's highest melanoma burden while pioneering innovative prevention strategies that have become international models. The region's cancer profile reflects both the challenges posed by geographic and demographic factors beyond immediate control and the successes possible through sustained public health commitment.

Cancer affects approximately 250,000 people annually across Australia and New Zealand, with distinct patterns emerging between men and women. The overall cancer profile shows breast cancer leading at 10.3% of all diagnosed cases, followed by prostate (8.8%), colorectal (8.4%), melanoma (7.8%) and lung cancer (6.5%). It is estimated that approximately 2 in 5 people (41%) will be diagnosed with cancer by age 75. However, these statistics tell only part of the story—when non-melanoma skin cancers are included, skin cancer becomes by far the most common malignancy affecting both men and women across the region.

Gender-specific patterns reveal important differences in cancer risk. Among women, breast cancer dominates at 22.7% of diagnosed cases, followed by colorectal (8.8%), lung (6.3%) and melanoma (6.3%). Men face a different hierarchy, with prostate cancer representing 16.1% of cases, melanoma (9.1%), colorectal (8.2%) and lung cancers (6.6%). The mortality picture shifts dramatically from incidence patterns, with lung cancer claiming the predominating all cancer deaths, followed by colorectal, prostate, pancreatic and breast cancers. This mortality profile underscores lung cancer's particularly lethal nature despite advances in treatment.

In 2022, cutaneous melanoma accounted for approximately 331,700 cancer cases worldwide, with ultraviolet radiation identified as the major risk factor. In Australia, New Zealand, Northern Europe and North America more than 95% of melanoma cases are attributable to UV radiation exposure, with the highest attributable age-standardised rates occurring in regions with lighter-skinned populations. Australia and New Zealand hold the unfortunate distinction of reporting among the world's highest melanoma incidence rates at approximately 37 and 30 cases per 100,000 people, respectively—making melanoma responsible for 8% of all cancer cases in the region. This rate dramatically exceeds other high-incidence countries, with the United States, and the UK reporting roughly half the incidence at 16.5 and 15.3 per 100,000. The region's extreme melanoma burden results from a perfect storm of environmental and demographic factors: geographic latitude that receives intense UV radiation, combined with a predominantly fair-skinned population of largely Celtic origin.

Australia has pioneered comprehensive skin cancer prevention strategies that offer valuable lessons for global cancer control. Government-sponsored education campaigns focused on preventing sunburn and reducing skin cancer risk, combined with widespread screening programs implemented since the 1980s, have been associated with reduced melanoma incidence and death rates across most population segments. These sustained efforts have successfully reduced both melanoma incidence and death rates across most population groups, though men over 70 continue to show increasing rates. Australia's approach to artificial UV exposure represents the most aggressive regulatory response globally. Research revealed that sunbeds caused 16% of all melanomas in the 18–29 age group, with 76% of young melanoma patients having used sunbeds previously. Australia is the only country that has implemented a complete ban on tanning beds for individuals of all ages, while other nations have limited restrictions to minors only. Australia's pioneering legislation banning tanning beds demonstrates how bold policy interventions can address emerging cancer risks before they become entrenched public health problems. As the global community grapples with rising cancer burdens, the Australia-New Zealand model provides a blueprint for how sustained commitment to evidence-based prevention can transform population health outcomes, even in the face of seemingly insurmountable environmental challenges.

## Transforming cancer from tragedy to a preventable crisis

Cancer prevention represents one of modern medicine's most powerful yet underutilised weapons, offering extraordinary potential to slash human suffering while generating massive healthcare savings. The case for prevention rests on a fundamental and sobering reality: at least half of all cancers could be prevented or diagnosed at an early, potentially curable stage using knowledge we already possess today. This evidence transforms our entire concept of cancer—from an inevitable tragedy that strikes without warning to a largely preventable crisis demanding coordinated global messaging leading to action.

The numbers tell a compelling story. According to new data in The Cancer Atlas, Fourth Edition (2025), an estimated 50% of cancer deaths worldwide stem from modifiable risk factors that we can control: tobacco and alcohol use, infections, excess body weight, poor diet, physical inactivity, ultraviolet radiation, environmental pollutants and workplace exposures. The tobacco epidemic alone accounts for over 20% of all cancer deaths globally, while infections drive 12% of new diagnoses. Excess body weight has been definitively linked to at least 13 cancer types and is associated with 40% of uterine cancer deaths and 20% of kidney cancer fatalities. These are not abstract statistics—they represent millions of lives that could be saved through targeted prevention strategies that we know how to implement.

### Preventing cancer: evidence-based strategies for global implementation

The case for cancer prevention spans from powerful individual interventions to transformative population health programs. Cessation of tobacco smoking stands out as the single greatest opportunity for cancer prevention worldwide, capable of preventing millions of deaths annually. The impact is both dramatic and measurable: quitting smoking achieves a 62% reduction in lung cancer deaths, while tobacco use remains the leading preventable cause of cancer across all demographics. This single intervention represents the clearest pathway to massive cancer burden reduction available to global health systems.

Several cancers can be virtually eliminated through existing medical interventions that achieve extraordinary effectiveness. Cervical cancer exemplifies this potential most powerfully. While the disease continues to devastate women across the developing world, screening programs alone can reduce mortality by 95%. HPV vaccination offers the potential to eliminate cervical cancer deaths entirely—a complete prevention success against a disease that is nearly 100% preventable. The tragedy lies in the thousands of women who continue to die annually from a condition that medical science has rendered almost entirely avoidable.

For those with genetic predispositions, medical interventions offer risk reductions that seemed impossible just decades ago. Women with high-risk genetic inheritance can achieve over 95% breast cancer risk reduction through prophylactic mastectomy, while endocrine chemoprevention cuts breast cancer incidence by 50% in appropriate candidates. BRCA1 and BRAC2 pathogenic variant carriers can reduce ovarian cancer risk by over 90% through prophylactic surgery and achieve similar breast cancer protection. These represent precision prevention at its most powerful—near-complete protection for those who need it most.

Liver cancer, the world's sixth most common cancer and third-leading cause of cancer death with 870,000 new cases annually, offers another prevention triumph. Since hepatitis B infection drives over half of all liver cancer cases worldwide, hepatitis B vaccination achieves a remarkable 90% mortality reduction. Combined with *H. pylori* treatment for stomach cancer prevention, these interventions demonstrate how targeting infectious causes can eliminate major cancer burdens. Even colorectal cancer shows extraordinary prevention potential, with colonoscopy screening reducing mortality by 53% through early polyp detection and removal.

Lifestyle-based prevention opportunities are equally compelling, particularly in confronting the obesity pandemic. Severe obesity now affects 43% of adults globally—more than 2.5 billion people—directly increasing risk for over a dozen cancer types, including breast, gastrointestinal, gynecological and thyroid cancers. In the United States alone, excess body weight drives more than 20% of all cancers. This crisis creates substantial prevention opportunities through diet, physical activity and comprehensive lifestyle programs. Emerging research on GLP-1 receptor agonists suggests these weight-loss medications may offer additional cancer prevention benefits.

The global cancer surge increasingly links to dietary shifts, particularly widespread consumption of processed and ultra-processed foods. High intake of processed meats, fried foods, sweets and heavily salted products independently increases risk for multiple cancers, including stomach cancer. These findings underscore nutrition's critical role as a modifiable cancer prevention factor and demand stronger public health strategies to reduce exposure to dietary carcinogens.

### The economic imperative for prevention interventions

The economic case for cancer prevention rivals the health benefits, representing one of modern healthcare's most cost-effective interventions. Cancer screening and early detection deliver extraordinary investment returns through multiple economic mechanisms extending far beyond immediate treatment savings. Early-stage cancer patients face treatment costs 2–4 times lower than late-stage diagnoses, while Medicare beneficiaries diagnosed at advanced stages encounter up to 7 times higher costs—a reality demonstrated across 500,000 beneficiaries studied.

These dramatic cost differentials reflect early-stage cancers' reduced intervention intensity, shorter treatment duration and fewer complications compared to advanced disease. The economic benefits ripple throughout society, reducing productivity losses through fewer work absences and disability claims while lowering indirect costs, including uncovered personal expenses. Patients diagnosed early achieve improved outcomes enabling fast return to normal activities, creating beneficial economic effects extending far beyond healthcare systems. Screening asymptomatic individuals maximises both health outcomes and cost-effectiveness by detecting disease before symptoms develop and treatment becomes complex and expensive.

### The European cancer screening index: a benchmark for progress

Current estimates suggest that at least 40% of European cancers could be prevented through effective implementation of existing knowledge about risk and protective factors. When combined with screening, early detection and medical prevention strategies, Europe could additionally avoid over one-third of cancer deaths by 2050—a potential reduction that would save hundreds of thousands of lives and billions of euros.

The launch of the European Cancer Screening Policy Index represents a watershed moment in Europe's systematic approach to cancer prevention. As the cornerstone of the ‘Time to Accelerate for Cancer Screening’ campaign, this evidence-based tool provides unprecedented insight into the state of cancer screening policies across EU Member States, creating accountability through transparent benchmarking and highlighting critical disparities that demand immediate attention.

Presently, only a select few European countries have fully implemented all recommended cancer screening programs covering breast, cervical, colorectal, prostate and lung cancers. Slovenia, Portugal, and Norway have emerged as continental leaders, demonstrating exemplary alignment with EU recommendations and serving as models for other nations to emulate. All EU member states except Bulgaria, Greece and the Slovak Republic have established breast cancer screening programs. The Nordic countries demonstrate exceptional performance, with Denmark achieving 83.0% participation, Finland 82.2% and Sweden 80.0%. Malta (77.8%) and Slovenia (77.2%) round out the top performers, illustrating that excellence is not limited to large, wealthy nations.

Twenty-two EU member states maintain cervical cancer screening programs using traditional Pap tests, while a progressive cohort including Denmark, Finland, Italy, Sweden, Romania and Portugal has pioneered the integration of HPV testing. In Bulgaria, Romania and some Baltic states, rising persistent HPV infection rates coincide with absent or inadequate HPV vaccination programs, creating conditions for increasing preventable cervical cancer. France presents a particularly concerning case, with HPV vaccination rates among young girls reaching only 25%—a public health failure in one of Europe's most developed nations.

The European Cancer Screening Policy Index provides the framework for accountability and progress measurement. Now Europe must demonstrate the political will to transform its cancer prevention potential into reality, ensuring that geographic location no longer determines cancer survival prospects across the continent.

## Universal genetic testing and pharmacogenomics in cancer care

Cancer care stands at a pivotal moment in medical history. After decades of fighting disease after it strikes, we now possess the genetic tools to predict, prevent and precisely treat many cancers before it claims lives. The convergence of rapidly advancing genetic science with decreasing costs has created an unprecedented opportunity: universal genetic testing and pharmacogenomics could fundamentally transform cancer care from reactive treatment to proactive prevention. While this technology currently flourishes primarily in resource-rich nations, the benefits are so compelling that global adoption appears inevitable as costs plummet and accessibility expands.

The genetic reality of cancer has fundamentally dismantled our previous assumptions about hereditary risk. Among patients already battling cancer, the prevalence of inherited predisposition is staggering: 25% of ovarian cancer patients carry hereditary variants, as do 10% of those with breast, colorectal, prostate and pancreatic cancers. These are not merely statistics—they represent millions of families whose medical destinies could be rewritten through early genetic identification and intervention.

Consider the life-altering implications for unaffected family members. Women carrying BRCA1 mutations face a devastating 30%–40% lifetime risk of developing ovarian cancer, while BRCA2 carriers confront a 20% risk. These numbers represent a ticking genetic time bomb that precision medicine can defuse. When hereditary risk is identified before cancer develops, prophylactic surgeries, enhanced screening protocols and targeted lifestyle modifications can dramatically reduce cancer incidence, transforming genetic knowledge from abstract information into concrete life-saving action.

In addition to known pathogenic variants, the current era of genetic testing now incorporates polygenic risk scores (PRS), which can identify individuals at very high genetic risk through the combined effects of hundreds of low-risk genes acting in concert. The future of genetic testing will inevitably shift toward adopting PRS as a standard method for stratifying risk in the general population. When this genomic information is combined with family history, lifestyle factors (including smoking, obesity and exercise), age and gender, it can provide personalised and highly specific cancer risk assessments for individuals. This comprehensive risk profiling enables people to engage in tailored prevention strategies and screening protocols that may extend beyond standard guidelines for their age-matched peers.

However, our current approach to genetic testing exposes a dangerous blind spot that may be costing lives. Traditional clinical criteria for determining who are recommended to receive genetic evaluation are proving woefully inadequate. A groundbreaking study of 361 colorectal cancer patients revealed that while 15.5% carried pathogenic mutations, our standard clinical guidelines would have missed 25% of these carriers entirely. This finding illuminates a critical shortfall in our medical system: we are systematically overlooking one in four hereditary cancer cases simply because they do not conform to our conventional risk assessment models.

The true scope of hereditary cancer emerges when we examine broader cancer populations rather than relying on selective testing. Among 17,523 cancer patients who received comprehensive genetic testing, a remarkable 16.7% carried hereditary cancer pathogenic variants—a proportion that fundamentally challenges the medical community's long-held assumption that hereditary cancer represents a rare phenomenon. More critically, these data expose a troubling gap: many individuals with actionable genetic variants would never have qualified for testing under traditional guidelines, remaining unaware of their hereditary risk until after cancer struck.

This mounting evidence is reshaping medical practice at the highest levels. In 2024, the American Society of Clinical Oncology and the Society of Surgical Oncology made a landmark policy shift, recommending genetic testing for nearly all breast cancer patients—a dramatic departure from the restrictive, criteria-based approaches that have dominated for decades. This evolution reflects a growing recognition that comprehensive testing identifies significantly more at-risk individuals and their families, ultimately preventing more cancers than selective strategies ever could. International data support this transformation: in Singapore, one-third of 1,154 cancer patients carried pathogenic germline variants that not only informed family risk but enabled many with advanced cancer to access targeted, germline-directed therapies.

The implications extend far beyond cancer patients to the broader healthy population. In a groundbreaking genomics study of 354,957 participants from a single U.

S. health system, 1 in 30 individuals (3.3%) harbored potentially actionable genetic findings. However, nearly 90% remained completely unaware of their elevated risk prior to screening—a stunning revelation about hidden genetic vulnerabilities throughout the population. Another large-scale U.S. screening study found that whole genome sequencing identified clinically significant inherited variants in 7.8% of participants: 3% carried cancer predisposition genes, 3.5% had reproductive condition variants and 1.3% possessed cardiovascular disease genes. Some individuals carried multiple high-risk variants, compounding their genetic vulnerability.

This shift toward universal testing represents more than diagnostic expansion—it transforms genetic assessment from a reactive tool deployed after cancer diagnosis into a proactive prevention strategy capable of intercepting disease before it develops. Multi-gene panel testing identifies actionable pathogenic variants that enable clinicians to implement risk-reduction strategies for new primary cancers, fundamentally altering the trajectory from treatment to prevention.

Perhaps most compelling is genetic testing's power to motivate the behavioural changes that traditional health education has struggled to achieve. When women learn about their genetic risk for breast and ovarian cancer, 72% report they ‘would try harder to have a healthy lifestyle.’ This statistic reveals precision medicine's untapped potential as a behaviour change catalyst, suggesting that personalised genetic risk information may prove to be among the most powerful tools available for motivating the lifestyle modifications essential to cancer prevention. In an era where population-wide health messaging often falls on deaf ears, individual genetic insights may finally provide the personalised motivation needed to transform prevention from concept into action.

### Pharmacogenomics: optimising treatment while minimising harm

While genetic testing addresses cancer prevention, pharmacogenomics confronts an equally critical challenge: ensuring that life-saving treatments do not become life-threatening. The fundamental flaw in modern prescribing should surprise no one—humans do not metabolise drugs at the same rate. However, standard drug dosing persists with a traditional ‘one size fits all’ approach, creates a medical lottery where some patients metabolise medications too rapidly to receive any benefit, while others process them so slowly that standard doses become toxic.

Pharmacogenomics transforms drug prescribing—for both everyday medications and cancer therapies—from educated guesswork into a more precision science. While this genetic testing approach has not yet reached widespread clinical application, it represents an important emerging technology. By integrating genetic profiles with clinical, social and administrative data, pharmacogenomics maximises therapeutic benefits while preventing the devastating side effects that have long been accepted as inevitable collateral damage of treatment. In a healthcare system where the right drug at the wrong dose can mean the difference between cure and catastrophe, pharmacogenomics offers a path from crude population averages to individualised precision, ensuring that each patient receives exactly what their genetic makeup demands for optimal metabolism of their prescribed drug.

The urgency for this transformation becomes starkly apparent when examining medication-related harm across America. Adverse drug reactions rank as the fourth leading cause of death nationwide, while suboptimal medication therapy drains an estimated $528 billion annually from the healthcare system. In oncology, where therapeutic windows are razor-thin and toxicities can prove fatal, the stakes of precision prescribing reach their absolute peak.

Recent population screening studies reveal pharmacogenomics' transformative potential hiding in plain sight. In a comprehensive U.

S. healthcare system study involving whole genome sequencing, 2,017 individuals received management recommendations related to pharmacogenomics. The results were striking: every patient carried at least one genetic variant that could affect their current or future medication response, with 14.6% harboring variants requiring immediate dose adjustments for optimal safety and efficacy.

Parallel findings from the United Kingdom amplify this global pattern. Among 76,805 participants, 5.2% carried clinically relevant pharmacogenetic variants associated with drug-induced toxicity across just four key genes. The implications are profound: approximately 14,540 cancer patients annually could benefit from reduced doses of antimetabolite chemotherapy drugs or alternative medications, potentially preventing thousands of severe adverse reactions.

The China National Genomics Pilot provides the most compelling real-world validation of pharmacogenomics in cancer care. This landmark implementation study enrolled over 6,000 adult oncology patients across 11 tertiary cancer hospitals, focusing on DPYD-guided dosing for fluoropyrimidine-based chemotherapy. Patients with DPYD variants metabolise these drugs differently, making standard dosing potentially toxic or even lethal. The results were nothing short of revolutionary: genetic-guided prescribing reduced toxicity-related hospitalisations by 45%, with each patient saving approximately $1,450 in avoided hospitalisation, laboratory and testing costs.

These outcomes demonstrate that pharmacogenomics has evolved beyond theoretical promise into near-term future reality. The dramatic reduction in toxicity-related hospitalisations can potentially translate into fewer treatment delays, dramatically improved quality of life and more efficient healthcare resource utilisation. Perhaps most compelling, the economic analysis reveals that pharmacogenomic testing essentially pays for itself through prevented complications and reduced healthcare consumption.

This emerging technology has the potential to transform drug prescribing—for both conventional medications and cancer therapeutics—from empirical trial-and-error approaches into precision science. By integrating genetic, clinical, social and administrative data, pharmacogenomics can maximise therapeutic benefits while minimising devastating side effects for the patients and saving substantial costs from toxicity management. This represents a fundamental paradigm shift in cancer treatment: moving from accepting toxic side effects as inevitable collateral damage to preventing toxicity through genetic foresight. As precision medicine continues to evolve, pharmacogenomics and ‘precision prescribing’ stand as compelling evidence that the future of cancer care lies not just in developing more powerful cancer treatments, but in deploying existing therapies more intelligently and safely.

## A moral imperative for precision cancer medicine

The transition to precision medicine requires a fundamental cultural shift in oncology practice. As technological and scientific advances become increasingly integrated into clinical care, genetic information—both the inherited germline profile and the somatic molecular profile of the tumour—will evolve from a specialised tool to an essential component of cancer treatment—as routine and indispensable as tumour staging or performance status assessment. This transformation demands comprehensive, ongoing education and training programs to ensure healthcare providers can confidently interpret genetic results, understand their clinical implications and translate complex genomic data into actionable treatment decisions that improve patient outcomes.

Patient education represents another critical component of successful implementation. Individuals must understand the value of genetic testing, not only for their own care but also for their families' future health. This education should address concerns about genetic discrimination while emphasising the preventive and therapeutic benefits of genetic knowledge.

The convergence of evidence supporting universal genetic testing and pharmacogenomics in cancer care creates a compelling case for fundamental changes in oncology practice. We now possess the tools to identify individuals at high risk for cancer before disease develops, enabling preventive interventions that can save lives. Simultaneously, we can optimise cancer treatments based on individual genetic profiles, improving outcomes while reducing toxicity and healthcare costs.

The question is no longer whether we should implement universal genetic testing and pharmacogenomics, but how quickly we can overcome the barriers to widespread adoption. Every day we delay comprehensive implementation, we miss opportunities to prevent cancers in high-risk individuals and to optimise treatments for those already diagnosed. The technology exists, the evidence is clear and the benefits are substantial—both for individual patients and for healthcare systems.

The transformation of cancer prevention from theory to practice requires recognition that prevention represents not just a health opportunity but an economic imperative. With treatment costs escalating and cancer incidence projected to increase dramatically over the coming decades, prevention strategies offer the possibility of bending the cost curve while saving lives. However, these advances bring potential challenges, including risks of genetic discrimination, insurance complications, strained familial relationships and clinical uncertainty in risk assessment.

To realise prevention's full potential, policymakers and insurance companies must establish robust legal frameworks and policies that protect the confidentiality of genetic information and ensure that risk profiles are not used in discriminatory ways. When properly implemented with appropriate safeguards, the evidence demonstrates that investing in prevention yields extraordinary returns—both in human lives saved and healthcare costs avoided. This makes comprehensive cancer prevention programs among the most critical and cost-effective investments any healthcare system can make.

## A call to action

Looking toward the future, these projections reveal a world facing a cancer crisis of unprecedented scale of death, suffering and economic burden—where incidence nearly doubles within 25 years while half of all cancers remain preventable or curable if detected early. Without decisive intervention, this burden could devastate those communities least equipped to respond, driving millions of families into financial ruin and deepening global health inequities and economies in nations least equipped to afford it. The data demand urgent action from policymakers, healthcare systems and the international community to prevent cancer from becoming an even greater driver of global inequality.

However, this crisis is not inevitable. In fact, millions of lives could be saved using the knowledge and interventions we already possess. The barrier is not scientific understanding but implementation at scale. Reducing the burden of chronic diseases and cancer requires moving upstream to tackle root causes. We must address shared risk factors and social determinants of health, ensure equitable prevention and screening across all life stages, and build partnerships that span sectors. This comprehensive approach is essential to confronting these closely linked health challenges. To accomplish these ambitious and long-term goals, we need coordinated global action: governments investing in prevention funding and infrastructure, healthcare systems prioritising early detection, insurance systems covering proven interventions and communities mobilising around evidence-based strategies that work.

While our passion for reducing the global cancer burden drives important progress, significant challenges remain. Targeted initiatives have enhanced cancer care delivery in high-resource countries, but fundamental barriers remain in low-resource countries: insufficient public awareness, deeply rooted societal stigma, fragmented healthcare infrastructure and critical shortages of oncology and surgical specialists. We urgently need more research to develop effective interventions and cost-effective prevention strategies for general populations, alongside better management approaches for cancer patients, regardless of where they live. Our understanding of communities bearing the heaviest disease burden remains inadequate.

This crisis, while daunting in scope, is not inevitable. The patterns revealed in these projections represent a call to action—an opportunity to reshape cancer's future impact through coordinated global effort, strategic investment in prevention and early detection and a commitment to ensuring that geography and economic status no longer determine whether a cancer diagnosis becomes a death sentence or a manageable medical condition.

## Conflicts of interest

None known.

## Funding

None.

## Footnotes

* For more details by country, go to www.iarc.who
